# Deep Learning in Heart Sound Analysis: From Techniques to Clinical Applications

**DOI:** 10.34133/hds.0182

**Published:** 2024-10-09

**Authors:** Qinghao Zhao, Shijia Geng, Boya Wang, Yutong Sun, Wenchang Nie, Baochen Bai, Chao Yu, Feng Zhang, Gongzheng Tang, Deyun Zhang, Yuxi Zhou, Jian Liu, Shenda Hong

**Affiliations:** ^1^ Department of Cardiology, Peking University People’s Hospital, Beijing, China.; ^2^ HeartVoice Medical Technology, Hefei, China.; ^3^ Key Laboratory of Carcinogenesis and Translational Research (Ministry of Education/Beijing), Department of Gastrointestinal Oncology, Peking University Cancer Hospital and Institute, Beijing, China.; ^4^ National Institute of Health Data Science, Peking University, Beijing, China.; ^5^ Institute of Medical Technology, Health Science Center of Peking University, Beijing, China.; ^6^ Department of Computer Science, Tianjin University of Technology, Tianjin, China.; ^7^ DCST, BNRist, RIIT, Institute of Internet Industry, Tsinghua University, Beijing, China.

## Abstract

**Importance:** Heart sound auscultation is a routinely used physical examination in clinical practice to identify potential cardiac abnormalities. However, accurate interpretation of heart sounds requires specialized training and experience, which limits its generalizability. Deep learning, a subset of machine learning, involves training artificial neural networks to learn from large datasets and perform complex tasks with intricate patterns. Over the past decade, deep learning has been successfully applied to heart sound analysis, achieving remarkable results and accumulating substantial heart sound data for model training. Although several reviews have summarized deep learning algorithms for heart sound analysis, there is a lack of comprehensive summaries regarding the available heart sound data and the clinical applications. ** Highlights:** This review will compile the commonly used heart sound datasets, introduce the fundamentals and state-of-the-art techniques in heart sound analysis and deep learning, and summarize the current applications of deep learning for heart sound analysis, along with their limitations and areas for future improvement. ** Conclusions:** The integration of deep learning into heart sound analysis represents a significant advancement in clinical practice. The growing availability of heart sound datasets and the continuous development of deep learning techniques contribute to the improvement and broader clinical adoption of these models. However, ongoing research is needed to address existing challenges and refine these technologies for broader clinical use.

## Introduction

**Fig. 1.**
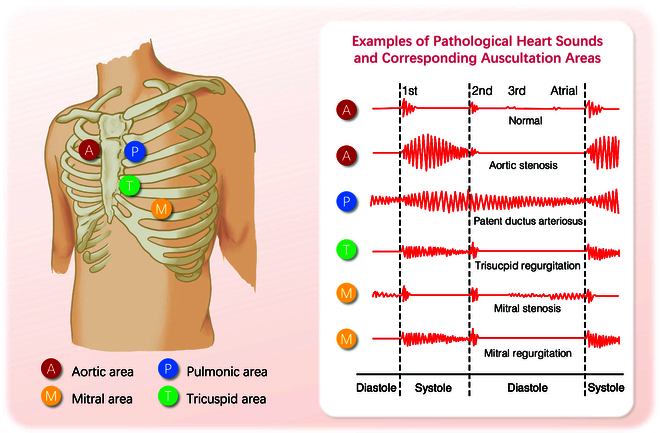
Auscultation locations and example of pathological heart sounds. Reproduced under the CC BY-SA 3.0 license and adapted from Madhero [162]

During the cardiac cycle, the mechanical contractions of the heart, along with the opening and closing of the heart valves, propel blood between the heart chambers and throughout the body. This mechanical activity and the turbulent motion of the blood flow cause vibrations in the heart and arterial structures [1]. These vibrations, i.e., heart sounds, are audible on the chest wall, and their graphical time series representation is known as phonocardiogram (PCG). Four locations are most often used to capture the heart sounds, named based on the positions where the valves are most clearly audible: aortic area, pulmonic area, tricuspid area, and mitral area (Fig. 1). When pathological changes occur in the structure and function of the heart, characteristic alterations in heart sounds manifest in specific auscultation areas. These sounds’ timing, shape, maximum intensity location, radiation, and frequency content are valuable features for distinguishing diseases (Fig. 1). For instance, aortic stenosis (AS) often presents with a characteristic rough systolic murmur, best heard at the right upper sternal border. This murmur typically has a crescendo–decrescendo pattern, increasing in intensity during mid-systole and then decreasing toward the end of systole. Additionally, the intensity and duration of the murmur can vary: a louder and longer murmur with a late peak suggests more severe stenosis [1].

Cardiac auscultation, the practice of listening to heart sounds with a stethoscope, is a noninvasive and easy-to-operate technique widely used by medical professionals to identify potential cardiac abnormalities. However, the interpretation of heart sounds can vary greatly depending on the experience and skill of the examiners. Even in detecting the systolic murmurs, which is a common task in auscultation, the reliability is mediocre at best (Cohen’s *k* for agreement measurement = 0.3 to 0.48) [2], and the ability to identify other pathological features is even worse [3]. In addition, the growing availability of recording techniques has led to a substantial accumulation of heart sound data, particularly long-term heart sound monitoring. However, manual analysis using traditional auscultation methods is no longer sufficient to handle the data surge, especially in real-time scenarios.

To address these challenges, researchers are actively developing computational methods for analyzing heart sound data. In recent years, the potential of deep learning (DL) techniques in PCG analysis has gradually attracted the interest of researchers. DL is suitable for tasks involving a large amount of data with complex patterns, and the PCG signal has a high sampling frequency and contains rich information in both the time and frequency domains, consistent with this data characteristic. DL models can be trained using either raw signals in the time domain or pre-processed frequency information, allowing them to capture subtle patterns in heart sounds that may be difficult for physicians to discern. By leveraging this underlying knowledge, these models have demonstrated remarkable diagnostic accuracy across a range of cardiac conditions [4,5]. Furthermore, DL-based heart sound diagnostic techniques offer a cost-effective and user-friendly approach, making them a more economical and accessible alternative for disease screening than traditional medical imaging techniques. This advantage is precious for undiagnosed patients in underdeveloped regions who may face limited access to specialized medical imaging equipment and trained healthcare providers.

In recent years, several reviews have been done on DL for heart sound analysis [6,7]. However, these reviews cover a relatively short time span (4 to 5 years) and lack the latest advancements (up to 2022), thus failing to provide a comprehensive overview of DL-based heart sound analysis. Additionally, previous reviews lack a thorough summary of publicly available databases, which are crucial for training DL models. This gap makes it challenging for researchers to utilize existing public resources effectively. Moreover, while these reviews primarily focus on DL algorithms used in heart sound analysis, such as denoising, segmentation, and model architecture, they fall short of summarizing the clinical applications of these technologies. This gap makes it challenging for non-computer specialists, such as medical staff, to stay updated on the latest advancements.

To fill these gaps, our review aims to provide a comprehensive overview of the field from 2010 to 2024. We will begin by summarizing commonly used datasets in heart sound analysis, covering both publicly available and private heart sound databases. Then, we will introduce the entire process of constructing a DL-based heart sound diagnostic model, detailing fundamental steps such as data pre-processing, feature extraction, and the deployment of DL models, along with recent advancements. In doing so, we will explain the principles of heart sound analysis and DL, highlighting why DL is an effective approach for this application. Additionally, we will provide an extensive summary of the clinical applications of DL-based heart sound analysis. Finally, we will discuss the current challenges and future perspectives in this field.

## Methods

### Search strategy

To summarize current research findings on heart sound analysis using DL, we searched PubMed, Embase, Web of Science, and Google Scholar for “deep learning” or “machine learning” or “artificial intelligence” in conjunction with “heart sounds” or “cardiac sounds” or “heart murmur” or “cardiac murmur” or "phonocardiogram” or “phonocardiography” or “PCG”, and covers the period from 2010 January 1 to 2024 January 1. All keywords are case-insensitive. To avoid missing papers that did not explicitly mention these keywords in their titles, we expanded our search to include all fields in each article. In total, 277 related studies were found.

### Study selection

We only included published peer-reviewed articles and excluded reviews, editorials, non-heart sound studies, and non-artificial intelligence studies. As this review focused on DL, we excluded those studies with a narrow sense of machine learning (ML) involved with conventional ML algorithms and applications outside the DL regime. However, we retained research on neural networks (NNs) and their variants because of their close proximity to DL model structures. In addition, we excluded literature written in a language other than English and with obvious errors in the results and methods sections.

**Fig. 2.**
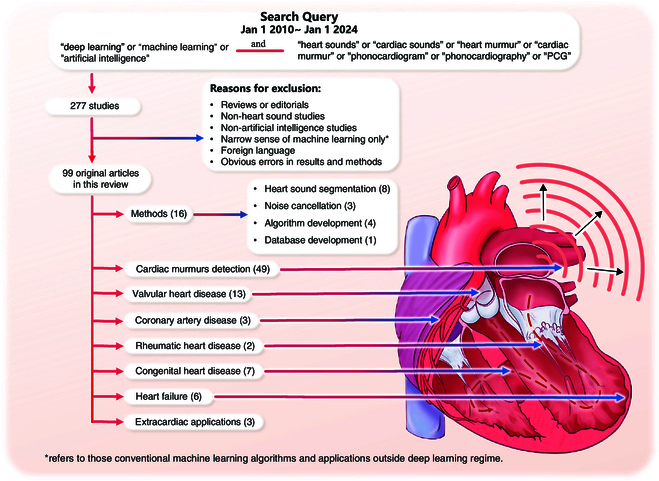
Diagram demonstrating the literature selection process and clinical applications of deep learning in heart sound analysis.

The process of literature searching and selection is illustrated in Fig. 2. At last, 99 original articles were included. These studies can be broadly categorized into several groups: methods (16 papers, including heart sound segmentation, noise cancellation, algorithm development, and database development), cardiac murmurs detection (49 papers), valvular heart disease (VHD; 13 papers), congenital heart disease (CHD; 7 papers), heart failure (HF; 6 papers), coronary artery disease (CAD; 3 papers), rheumatic heart disease (RHD; 2 papers), and extracardiac applications (3 papers, including blood pressure [BP] and pulmonary artery pressure [PAP] estimation).

## Results

This chapter begins by exploring the heart sound datasets, which serve as the foundation for DL modeling. We then delve into the fundamental techniques for heart sound analysis, detailing essential steps such as data pre-processing, feature extraction, and deploying DL models. Through this comprehensive examination, we present the entire process of constructing a DL-based heart sound diagnostic model, thereby contributing to our understanding of the field.

### Heart sound datasets

The study of sound is a discipline with ancient roots that can be traced back centuries. Before the invention of modern medical imaging techniques, physicians mainly relied on sounds to gain insights into the inner workings of the body, particularly heart sound auscultation. As recording technology has advanced, it has become possible to capture heart sounds in both analog and electronic forms, leading to the accumulation of vast amounts of data that can be utilized for in-depth analysis. Today, these sound files are typically saved in .mp3 or .wav formats, which can be loaded, visualized, and analyzed using Python libraries such as librosa and other audio processing tools. This technological process has facilitated the availability of publicly accessible datasets that serve as valuable resources for benchmarking and testing novel methods and approaches in heart sound analysis.

Table 1 presents a selection of well-known public heart sound datasets. The PASCAL [8], CinC2016 [9], and CinC2022 [10] have large sample sizes but lack annotations for specific diseases causing murmurs, making them ideal for training heart murmur detection models. The dataset by Yaseen et al. [11] specifically targets VHDs, including recordings of AS, mitral stenosis (MS), mitral regurgitation (MR), and mitral valve prolapse (MVP). Additionally, the HSS dataset [12] further provides a detailed classification of valvular disease severity, categorizing it into mild and moderate/severe grades based on echocardiographic assessments. The EPHNOGRAM dataset [13] is distinguished by its inclusion of simultaneous electrocardiogram (ECG) and heart sound recordings taken during fitness exercises. The SUFHSDB dataset [14] features both fetal and maternal heart sounds. Furthermore, the dataset by Gradolewski et al. [15] combines open data from 5 sources, offering a small yet heterogeneous collection of heart sound recordings.

**Table 1.** Public heart sound datasets

Dataset	Resource	Sampling frequency	Recording duration	Number of recordings and/or subjects (if mentioned)
PASCAL [8]	Two sources: (a) from the general public via the web and (b) from hospital patients	(a) 44,100 Hz	1 to 30 s	(a) 124 recordings (31 normal, 34 murmur, 19 extras, and 40 artifacts)
		(b) 4,000 Hz		(b) 461 recordings (320 normal, 95 murmur, and 46 artifacts)
CinC2016 [9]	9 databases from different research groups	2,000 Hz	5 to 120 s	3,153 recordings (2,488 normal and 665 abnormal with CAD or VHD)
CinC2022 [10]	2 mass pediatric screening campaigns conducted in Brazil	4,000 Hz	4.8 to 80.4 s	5,272 recordings from 1,568 subjects (486 normal subjects and 1,082 subjects with heart diseases)
HSS [12]	Patients with various health conditions from the hospital	4,000 Hz	30 s on average	845 recordings (144 normal, 465 mild, and 236 moderate/severe) from 170 subjects
Yaseen’s [11]	Books (auscultation skills CD, heart sound made easy) and 48 websites	8,000 Hz	3 to 5 s	1,000 recordings (200 for each: normal, AS, MS, MR, and MVP)
EPHNOGRAM [13]	Healthy adults, indoor fitness, simultaneous electrocardiogram, and phonocardiogram	8,000 Hz	30 s or 30 min	69 recordings (10 bicycle stress test, 11 treadmill, 13 static bike, and 35 resistance training) from 24 subjects
SUFHSDB [14]	Fetal and maternal heart sounds obtained at Hafez Hospital, Shiraz University	16,000 Hz; 44,100 Hz	90 s on average	119 recordings from 109 subjects
Gradolewski’s [15]	5 sources: Michigan, eGeneral Medical Inc., 3M Littmann, University of Washington, and Thinklabs	8,000 to 11,000 Hz	4 to 139 s	5 * 10 recordings

In addition to publicly available datasets, numerous researchers collected their heart sound data for specific research purposes. Table 2 provides a comprehensive overview of various private heart sound datasets and their applications. These datasets originate from diverse sources and cover various heart-related conditions, such as VHDs, HF, and CHDs. These datasets exhibit diversity in sampling frequency, spanning from 2,000 Hz to 8,000 Hz, and provide recordings of varying durations, ranging from short 5-s clips to extensive 120-s recordings. Their scale also varies significantly, from only dozens to thousands of subjects. Each dataset has been cited in one or more research studies, emphasizing their importance in advancing the understanding and analysis of heart sounds.

**Table 2.** Private heart sound datasets

Dataset	Application	Resource	Sampling frequency	Recording duration	Number of recordings and/or subjects (if mentioned)
Private heart murmur dataset, Chorba et al. (2021) [20]	Heart murmur detection	Patients from 4 echocardiography laboratories and structural heart disease clinics in the US	4,000 Hz	15 s × 4 positions	1,774 recordings (682 murmur and 1,092 normal) from 962 subjects
Multisite Eko dataset, Prince et al. (2023) [44]	Heart murmur detection	Multisite clinical studies	–	5 to 120 s	10,965 recordings
Private algorithm development dataset, Arjoune et al. (2023) [101]	Algorithm development	Children from Children’s National Hospital, US	–	15 s	470 recordings (265 Still’s murmur and 205 pathological heart murmur)
Private VHD dataset 1, Makimoto et al. (2022) [45]	Valvular heart disease	Patients at the Faculty of Medicine at Heinrich Heine University Düsseldorf, Germany	4,000 Hz	15 s × 3 positions	836 subjects (670 normal, 51 mild AS, 51 moderate AS, and 114 severe AS)
Private VHD dataset 2, Waaler et al. (2023) [58]	Valvular heart disease	From Tromsø7 study [163], Norway	44,100 Hz	10 s × 4 positions	800 recordings from 200 participants
Private VHD dataset 3, Shiraga et al. (2023) [46]	Heart failure	Patients at the University Hospital Düsseldorf, Germany	4,000 Hz	15 s × 3 positions	1,052 subjects
Private CHD dataset 1, Gharehbaghi et al. (2017) [91]	Congenital heart disease	Children referrals to the Children Medical Centre Hospital of Tehran University, Iran	–	10 s	90 subjects (30 VSD, 15 MR, 15 TR, and 30 normal)
Private CHD dataset 2, Wang et al. (2020) [36]	Congenital heart disease	Patients at the National Taiwan University Hospital	–	10 s × 5 positions × 2	776 recordings (525 VSD and 251 normal) from 76 subjects
Private CHD dataset 3, Liu et al. (2022) [21]	Congenital heart disease	Children admitted to Children’s Hospital of Chongqing Medical University, China	22,050 Hz	10 s × 5 positions	884 subjects (409 normal, 192 ASD, 98 VSD, 95 PDA, and 90 combined CHD)
Private CHD dataset 4, Gharehbaghi et al. (2020) [22]	Congenital heart disease	Children referrals to Tehran University of Medical Sciences, Iran	44,100 Hz	10 s	115 subjects (10 ASD, 25 innocent murmur, 15 MR, 15 TR, 25 VSD, and 25 normal)
Private CHD dataset 5, Huang et al. (2022) [102]	Congenital heart disease	Patients at the E-Da Hospital	8,000 Hz	10 s * 3 positions * 2	184 subjects (46 VSD, 50 ASD, and 8 normal)
Private CHD dataset 6, Hassanuzzaman et al. (2023) [23]	Congenital heart disease	Children from Bangladesh Shishu (Children) Hospital and Institute and National Heart Foundation Hospital and Research Institute, Bangladesh	4,000 Hz	15 s	2,068 recordings from 484 subjects (297 CHD and 187 non-CHD)
Private CHD dataset 7, Wang et al. (2023) [116]	Congenital heart disease	CHD screenings in various mountainous primary schools across Yunnan, China	5,000 Hz	20 s	133 synchronized heart sound—ECG recordings and 7,000 heart sound recordings
Private HF dataset 1, Gao et al. (2020) [18]	Heart failure	Patients at University-Town Hospital of Chongqing Medical University, China	11,025 Hz	–	108 subjects (42 HFrEF and 66 HFpEF)
Private HF dataset 2, Wang et al. (2022) [17]	Heart failure	Patients at the First Affiliated Hospital of Chongqing Medical University, China	4,000 Hz	3 min	136 subjects (59 HFrEF and 77 HFpEF)
Private HF dataset 3, Yang et al. (2021) [37]	Heart failure	Patients at the First Affiliated Hospital of Chongqing Medical University, China	8,000 Hz	5 min	71 subjects (30 LVDD and 41 normal)
Private HF dataset 4, Zheng et al. (2022) [19]	Heart failure	Patients at the First Affiliated Hospital and the University-Town Hospital of Chongqing Medical University, China	8,000 Hz	5 min	224 HF subjects (42 Stage A, 56 Stage B, 75 Stage C, and 51 Stage D), 51 normal
Private HF dataset 5, Chen et al. (2023) [77]	Heart failure	Patients at the First Affiliated Hospital of Chongqing Medical University, China	8,000 Hz	3 or 5 min	121 subjects (32 NYHA class II, 56 class III, and 33 class IV)
Private HF dataset 6, Zheng et al. (2023) [103]	Heart failure	Patients at the First Affiliated Hospital of Chongqing Medical University, China	4,000 Hz	5 min	122 subjects (55 diastolic dysfunction and 67 control)
Dan-NICAD trial 1 dataset, Winther et al. (2021) [164]	Coronary artery disease	Patients from the Danish study of the Non-Invasive Testing in Coronary Artery Disease (Dan-NICAD) trial 1	–	3 min (8 s holding breath × 4)	1,464 subjects (723 CAD-score ≤ 20 and 741 CAD-score > 20)
Private CAD dataset 1, Li et al. (2021) [59]; Li et al. (2020) [104]	Coronary artery disease	Patients at Shandong Provincial Qianfoshan Hospital, China	1,000 Hz	5 min	195 subjects (135 CAD and 60 non-CAD)
Private CAD dataset 2, Ainiwaer et al. (2023) [24]	Coronary artery disease	Patients at First Affiliated Hospital of Xinjiang Medical University, China	4,000 Hz	30 s × 9 positions	2,880 recordings from 320 cases
Private RHD dataset, Asmare et al. (2020) [47]	Rheumatic heart disease	Patients at Tikur Anbessa Referral Teaching Hospital, College of Health Sciences, Addis Ababa University, Ethiopia	44,100 Hz	–	170 subjects (124 RHD and 46 normal)
Proposed RHD dataset, Ali et al. (2021) [25]	Rheumatic heart disease	Children from a group of schools serving the underprivileged in Karachi, Pakistan	–	–	Aim to recruit 1,700 children (definite RHD, subclinical RHD, and normal)
Private blood pressure dataset, Kapur et al. (2019) [151]	Blood pressure measurement	Critically ill children undergoing continuous blood pressure monitoring at the Children’s Hospital of Michigan/Wayne State University, US	–	>1 min × 2 positions simultaneously	25 subjects

### Heart sound analysis technologies

Applying DL techniques to heart sound analysis typically involves pre-processing heart sound data and deploying DL models, with feature extraction frequently included. In some cases, feature extraction and data pre-processing are integrated. In this section, we will summarize and discuss these stages.

#### Pre-processing and feature extraction

Since sound analysis has a long history of research and origin, heart sound analysis usually draws on techniques from traditional sound research for pre-processing and feature extraction.

In the pre-processing of heart sound analysis, traditional audio processing methods are applied, such as discrete wavelet transform smoothing [16], adaptive wavelet denoising [17], and Logistic Regression-Hidden Semi-Markov Model (LR-HSMM) segmentation [18,19]. These techniques help to improve the clarity and quality of the heart sound signal, thereby improving the accuracy of the subsequent analysis.

**Fig. 3.**
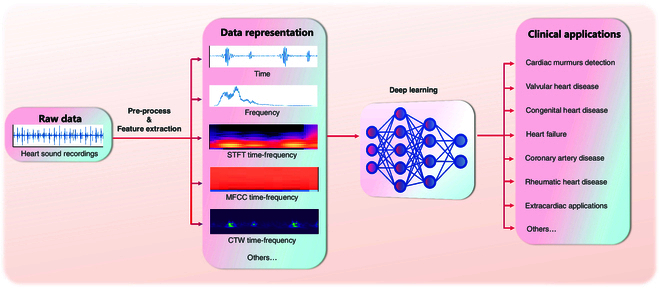
Different heart sound process pathways.

The input to a DL model can be either features extracted using a variety of time–frequency methods or a temporal representation that has undergone basic processing such as smoothing, denoising, and segmentation. The latter paradigm eliminates the need for a feature extraction step, allowing the model to process the raw data directly and deliver task-specific results in an “end-to-end” fashion [16–18,20–35]. Figure 3 illustrates the different process pathways.

With feature extraction step, traditional audio feature extraction techniques effectively capture the key characteristics of heart sound signals, which can then serve as inputs for DL models for further analysis. To extract frequency information from heart sounds, various techniques such as short-time Fourier transform (STFT) [36–43], Mel-spectrum [44–57], Mel-frequency cepstral coefficients (MFCCs) [58–76], and continuous wavelet transform (CWT) [15,77–85] are commonly utilized.

STFT, Mel-spectrum, and MFCCs are well-suited for general signal processing and audio analysis, offering simplicity and well-established methods. However, they come with fixed time–frequency resolutions, which can be a limitation when dealing with signals that have varying frequency content over time. For STFT, the signal is first split into short-time frames, assuming each frame contains stationary data. A window function is then applied to each frame, followed by a Fourier transform. The output of STFT is a 2-dimensional array of complex data, where the *x*-axis represents time with frame bins, and the *y*-axis represents the frequency contents ranging from zero to half of the sampling frequency. When visualized as a spectrogram, these values are displayed as the magnitude squared of the STFT values [86]. To obtain a Mel-spectrum [87], the power spectrum is first calculated, then Mel-scale triangular filters are applied to represent the spectrum power based on the characteristics of human hearing. MFCCs further compress the frequency representation by applying Discrete Cosine Transform (DCT) on the logarithm of the Mel-spectrum [88]. Unlike the aforementioned techniques that offer fixed resolution determined by the window size, CWT uses wavelets as basis functions to provide variable resolution. It delivers better frequency resolution at lower frequencies and better time resolution at higher frequencies, which makes CWT ideal for analyzing signals with nonstationary frequencies over time [89]. Other variable resolution time–frequency techniques include the complementary ensemble empirical mode decomposition (CEEMD) [19], a data-driven frequency analysis method; the tunable-Q wavelet transform (TQWT), which is derived from wavelet transform and offers variable time–frequency resolutions [19]; the spline kernel-based chirplet transform (SCT) [90], similar to wavelet transform but focused on capturing detailed variations in signals; and the growing length periodogram [22,91], which adjusts the window length as the analysis progresses. Both CEEMD and TQWT provide adaptive and flexible analysis suitable for nonlinear and nonstationary signals but come with higher computational costs and complexity. SCT offers detailed and localized time–frequency analysis, requiring careful parameter selection and significant computational resources. Similarly, the growing length periodogram demands extensive computational effort, especially as the window length increases.

#### Deep learning

DL, a subset of ML, involves training artificial NNs to learn from large datasets and perform complex tasks related to human cognitive activities and experiences. It has been successfully applied to various tasks, including image classification, speech recognition, natural language processing, and disease diagnosis [92–96]. DL models perform intricate functions based on large numbers of simple nonlinear computational units (known as artificial neurons) connected in complex hierarchical networks. This structure encourages each layer to learn simple representations that build up to sophisticated concepts. Compared to traditional ML, the fundamental architectural features of DL determine its greater ability to perform cohesive tasks, such as visual and computational knowledge representation. Another notable advantage of DL models is their ability to process raw data and automatically learn important features. Unlike traditional ML, which often requires handcrafted features, DL models can exploit some underlying features in raw data, enhancing classification accuracy and reducing the reliance on manual manipulation. However, as mentioned above, due to the maturity of sound processing techniques, feature extraction often remains an important part of performing sound-related tasks using DL.

The 2 most widely used DL model architectures are convolutional neural networks (CNNs) and recurrent neural networks (RNNs). CNNs are commonly used for grid-like data such as images and spectrograms. They employ multiple layers of convolutional filters to extract input features, followed by pooling layers to reduce the data dimensions. The output is then fed into fully connected layers for classification or regression tasks. RNNs, on the other hand, are designed to handle data with temporal dependencies, such as natural language and speech data. They process input sequences one unit at a time based on the current input unit and a hidden state that captures information from previous time steps.

In recent years, the Transformer architecture has gained significant popularity in DL. Initially developed for natural language processing, the Transformer has been adapted for various types of sequential input data, such as video, audio, and music [97–99]. Unlike RNNs, the Transformer can process the entire input sequence simultaneously using the attention mechanism, which allows it to focus on specific parts of the input sequence based on their relevance [100]. Consequently, the Transformer exhibits superior efficiency in both model training and inference stages.

Despite being one-dimensional signals, heart sounds pose challenges for traditional processing and analysis techniques due to their high sampling frequency and large number of samples per cardiac cycle. However, the emergence of DL models like CNNs, RNNs, and Transformers has made it possible to build high-performance models for heart sound analysis. These DL models can identify specific features relevant to various cardiac conditions, enhancing diagnostic accuracy and speed compared to manual methods. Moreover, DL techniques facilitate monitoring long-term changes in heart sounds, making them a powerful tool for continuous cardiac health tracking. By analyzing the evolving patterns and trends in heart sounds, healthcare professionals can detect and respond to cardiac abnormalities in a timely manner.

In the reviewed literature, most studies use CNN models with 2 to 34 convolutional layers [16,17,20,24,26,31,32,34,37–44,46–49,52–55,57,59,62,64–68,70–73,75–77,79–82,84,91,101–113], which are usually equipped with rectified linear units, batch normalization, dropout, and pooling components, and some of the layers are linked by residual connections. Wang et al. [85] tested 10 different CNN models including GoogleNet, SqueezeNet, DarkNet19, ModileNetv2, Inception-ResNetv2, DenseNet201, Inceptionv3, ResNet101, NasNet-Large, and Xception to compare the performances. Since heart sounds are continuous sequence signals, it is also suitable to test RNN models [22,56,58,61,114], while other studies combine both CNN and RNN [18,25,36,51,69,115]. Other models include Transformer or attention mechanism [23,78,116,117], traditional NN, and some NN modifications such as time delay NN, time growing neural network (TGNN), and kernel sparse representation network. Table 3 lists the heart sound analysis models in the reviewed articles.

**Table 3.** Data representations and models in heart sound analysis

Task class	Citation	Dataset	Data representation	DL (or NN) model
Heart sound segmentation	Martins et al. (2023) [118]	CinC2016, CinC2022	MFCC time–frequency, time feature	CNN (3 conv layers), HMM
Heart sound segmentation	Oliveira et al. (2021) [60]	CinC2016	MFCC time–frequency	RNN (GRU); CNN (3 conv layers)
Heart sound segmentation	Wang et al. (2021) [78]	CinC2016	CWT time–frequency	Transformer–CNN (3 conv layers)–RNN
Heart sound segmentation	Fernando et al. (2020) [114]	CinC2016	MFCC time–frequency, CWT time–frequency, frequency	RNN (LSTM)
Heart sound segmentation	Renna et al. (2019) [79]	CinC2016	CWT time–frequency, time	CNN
Heart sound segmentation	Meintjes et al. (2018) [80]	CinC2016	CWT time–frequency	CNN (3 conv layers)
Heart sound segmentation	Messner et al. (2018) [61]	CinC2016	MFCC time–frequency	RNN
Heart sound segmentation	Chen et al. (2017) [62]	Private	MFCC time–frequency	CNN
Noise cancellation	Marzorati et al. (2022) [26]	Private	Time	CNN (6 conv layers)
Noise cancellation	Tsai et al. (2020) [38]	Private	STFT time–frequency	CNN (33 conv layers)
Noise cancellation	Gradolewski et al. (2019) [15]	Gradolewski’s	CWT time–frequency	Time delay NN
Algorithm development	Yang et al. (2023) [48]	Yaseen’s	Mel time–frequency	CNN (U-net)
Algorithm development	Bao et al. (2022) [63]	CinC2016	MFCC time–frequency	CNN; RNN
Algorithm development	Soni et al. (2021) [39]	CinC2016	STFT time–frequency	CNN (17 conv layers)
Algorithm development	Gharehbaghi and Babic (2018) [35]	Private	Time	Deep time growing NN
Database development	Oliveira et al. (2022) [10]	CinC2022	–	–
Murmur detection	Prince et al. (2023) [44]	Private	Mel time–frequency	CNN (11 conv layers)
Murmur detection	Barnawi et al. (2023) [49]	CinC2016	Mel time–frequency	CNN
Murmur detection	Song et al. (2023) [64]	CinC2016	MFCC time–frequency	CNN
Murmur detection	Tsai et al. (2023) [65]	CinC2016	MFCC time–frequency	CNN (capsule)
Murmur detection	Xu et al. (2023) [66]	CinC2022	MFCC time–frequency	CNN
Murmur detection	Ma et al. (2023) [27]	CinC2016	Time	CNN (3 conv layers) + attention
Murmur detection	Gharehbaghi et al. (2023) [50]	CinC2016	Mel time–frequency	CNN–RNN
Murmur detection	Maity and Saha (2023) [105]	CinC2016	CWT time–frequency, STFT time–frequency	CNN (8 conv layers)
Murmur detection	Gharehbaghi et al. (2023) [51]	CinC2016	Mel time–frequency	CNN (parallel) + RNN
Murmur detection	Liu et al. (2023) [165]	CinC2016; CinC2022	Time–frequency	CNN (2 conv layers)–RNN–Attention
Murmur detection	Arjoune et al. (2023) [101]	Private	Time–frequency	CNN (3 conv layers)
Murmur detection	Han et al. (2023) [52]	CinC2022	Mel time–frequency	CNN (9 conv layers)
Murmur detection	Tariq et al. (2022) [106]	PASCAL	STFT time–frequency, MFCC time–frequency, chromagram	CNN
Murmur detection	Li et al. (2022) [107]	CinC2016	Mel time–frequency, MFCC time–frequency	CNN
Murmur detection	Zhu et al. (2022) [67]	CinC2016	MFCC time–frequency	CNN
Murmur detection	Zhou et al. (2022) [53]	CinC2016	Mel time–frequency	CNN (2 conv layers)
Murmur detection	Gharehbaghi and Babic (2022) [28]	Private	Time	CNN; deep time growing NN
Murmur detection	Tseng et al. (2021) [108]	CinC2016	Time, frequency, MFCC time–frequency	CNN (large size)
Murmur detection	Koike et al. (2021) [40]	CinC2016	STFT time–frequency	CNN
Murmur detection	Duggento et al. (2021) [68]	CinC2016	MFCC time–frequency	CNN
Murmur detection	Megalmani et al. (2021) [69]	CinC2016	MFCC time–frequency, time	CNN–RNN (LSTM)
Murmur detection	Bondareva et al. (2021) [166]	PASCAL	Time, frequency	DNN
Murmur detection	Ho et al. (2021) [81]	CinC2016	CWT time–frequency	CNN (2 conv layers)
Murmur detection	Duggento et al. (2021) [70]	CinC2016	MFCC time–frequency	CNN
Murmur Detection	Boulares et al. (2021) [71]	CinC2016; PASCAL	MFCC time–frequency	CNN
Murmur detection	Khan et al. (2021) [41]	CinC2016; PASCAL	STFT time–frequency	CNN
Murmur detection	Huai et al. (2021) [54]	Private; CinC2016	Mel time–frequency	CNN (3 conv layers)
Murmur detection	de Campos Souza (2020) [29]	CinC2016	Time	NN
Murmur detection	Dissanayake et al. (2020) [72]	CinC2016	MFCC time–frequency	CNN (3 conv layers)
Murmur detection	Koike et al. (2020) [55]	CinC2016	Mel time–frequency	CNN (12 conv layers)
Murmur detection	Deperlioglu et al. (2020) [30]	CinC2016; PASCAL	Time	NN
Murmur detection	Chen et al. (2020) [82]	CinC2016	CWT time–frequency	CNN (2 conv layers)
Murmur detection	Deng et al. (2020) [73]	CinC2016	MFCC time–frequency	CNN (3 conv layers), RNN
Murmur detection	Krishnan et al. (2020) [31]	CinC2016	Time	CNN (1 or 2 conv layers)
Murmur detection	Khan et al. (2020) [167]	CinC2016	MFCC time–frequency, time, frequency	NN; RNN (LSTM)
Murmur detection	Humayun et al. (2020) [109]	CinC2016	Time	CNN
Murmur detection	Han et al. (2019) [74]	CinC2016	MFCC time–frequency	Modified NN
Murmur detection	Thompson et al. (2019) [168]	Private	Time–frequency, time, frequency	Non-linear AI classifier
Murmur detection	Nogueira et al. (2019) [110]	CinC2016	MFCC time–frequency, time, frequency	CNN
Murmur detection	Sotaquirá et al. (2018) [111]	Private	Time–frequency, time, frequency	CNN (2 conv layers)
Murmur detection	Han et al. (2018) [75]	CinC2016	MFCC time–frequency	CNN (2 conv layers)
Murmur detection	Amiriparian et al. (2018) [56]	HSS	Mel time–frequency	RNN
Murmur detection	Humayun et al. (2018) [32]	CinC2016	Sub-band time–frequency	CNN (2 conv layers)
Murmur detection	Bozkurt et al. (2018) [112]	Private; CinC2016	Mel time–frequency, MFCC time–frequency, sub-band time–frequency	CNN
Murmur detection	Dominguez-Morales et al. (2018) [113]	CinC2016	Sub-band time–frequency	CNN
Murmur detection	Eslamizadeh and Barati (2017) [83]	PASCAL	CWT time–frequency	NN
Murmur detection	Kay and Agarwal (2017) [169]	CinC2016	CWT time–frequency, MFCC time–frequency, time, frequency	NN
Murmur detection	Maknickas and Maknickas (2017) [76]	CinC2016	MFCC time–frequency	CNN (2 conv layers)
Murmur detection	Gharehbaghi et al. (2014) [33]	Private	Time	Time growing NN
Valvular heart disease	Jamil and Roy (2023) [117]	Yaseen’s	MFCC time–frequency, LPCC time–frequency, CWT time–frequency	CNN; Transformer
Valvular heart disease	Ding et al. (2023) [84]	CinC2022	CWT time–frequency	CNN
Valvular heart disease	Roy et al. (2023) [124]	Yaseen’s	HHT time–frequency, time features, frequency features	CNN; RNN; CNN–RNN
Valvular heart disease	Waaler et al. (2023) [58]	Private	MFCC time–frequency	RNN
Valvular heart disease	Torre-Cruz et al. (2023) [42]	Yaseen’s	STFT time–frequency	CNN (2 conv layers)
Valvular heart disease	Ma et al. (2023) [34]	PASCAL	Time	CNN (3 conv layers)
Valvular heart disease	Shiraga et al. (2023) [46]	Private	Mel time–frequency	CNN (10 conv layers)
Valvular heart disease	Khan (2022) [43]	Yaseen’s dataset	STFT time–frequency	CNN (16 conv layers)
Valvular heart disease	Makimoto et al. (2022) [45]	Private	Mel time–frequency	Time growing NN
Valvular heart disease	Alkhodari and Fraiwan (2021) [16]	Yaseen’s dataset; CinC2016	Time	CNN (3 conv layers)
Valvular heart disease	Chorba et al. (2021) [20]	Private	Time	CNN (33 conv layers)
Valvular heart disease	Ghosh et al. (2020) [90]	Yaseen’s dataset; MHSMD	Chirplet transform time–frequency	Deep layer kernel sparse representation network
Valvular heart disease	Baghel et al. (2020) [57]	Yaseen’s dataset	Mel time–frequency	CNN (7 conv layers)
Congenital heart disease	Hassanuzzaman et al. (2023) [23]	Private	Time	CNN-Transformer
Congenital heart disease	Wang et al. (2023) [116]	Private	MFSC time–frequency	CNN-attention
Congenital heart disease	Liu et al. (2022) [21]	Private	Time	CNN–RNN; CNN; RNN
Congenital heart disease	Huang et al. (2022) [102]	Private	Bispectrum frequency	CNN
Congenital heart disease	Wang et al. (2020) [36]	Private	STFT time–frequency	CNN–RNN
Congenital heart disease	Gharehbaghi et al. (2020) [22]	Private	Time	RNN
Congenital heart disease	Gharehbaghi et al. (2017) [91]	Private	Time growing time–frequency	CNN (with TAP)
Heart failure	Chen et al. (2023) [77]	Private	CWT time–frequency	CNN
Heart failure	Zheng et al. (2023) [103]	Private	STFT time–frequency, MFCC time–frequency, S-transform time–frequency, gammatone time–frequency	CNN
Heart failure	Wang et al. (2022) [17]	Private	Time	CNN (3 conv layers)
Heart failure	Yang et al. (2022) [37]	Private	STFT time–frequency	CNN (multiple models)
Heart failure	Zheng et al. (2022) [19]	Private	CEEMD time–frequency, TQWT time–frequency	Deep belief network
Heart failure	Gao et al. (2020) [18]	Private; CinC2016	Time	CNN–RNN
Coronary artery disease	Ainiwaer et al. (2023) [24]	Private	Time	CNN
Coronary artery disease	Li et al. (2021) [59]	Private	Time, GAF 2D-frequency, MFCC time–frequency	CNN (1D with 10 conv layers; 2D with 3 conv layers)
Coronary artery disease	Li et al. (2020) [104]	Private	MFCC time–frequency	CNN (12 conv layers)
Rheumatic heart disease	Ali et al. (2021) [25]	Private	Time	CNN–RNN
Rheumatic heart disease	Asmare et al. (2020) [47]	Private	Mel time–frequency	CNN (5 conv layers)
Blood pressure	Kapur et al. (2019) [151]	Private	Time and frequency features	NN
Pulmonary hypertension	Mi et al. (2023) [115]	Private	Time features, frequency features	CNN-Bi-LSTM
Pulmonary hypertension	Wang et al. (2022) [85]	Private; Yaseen’s	CWT time–frequency	CNN (multiple models for transfer learning)

### Clinical applications

As shown in Fig. 2, aside from a handful of studies concentrating on method developments, such as segmentation [60–62,78–80,114,118], noise cancellation [15,26,38], algorithm development (cardiopulmonary sound separation [48], duration effect analysis [63], unlabeled data training [39], and model evaluation [35]), and database development [10], the majority of DL applications in heart sound analysis are related to the clinical field. This section outlines several clinical applications of DL in heart sound analysis and explores how DL is reshaping cardiovascular healthcare.

#### Cardiac murmurs detection

Detecting cardiac murmurs is a fundamental but crucial task in heart sound analysis. Cardiac murmurs are abnormal sounds produced during the cardiac cycle and can indicate underlying heart conditions. As mentioned previously, identifying heart murmurs through traditional auscultation is challenging because it requires considerable expertise and often results in variability among clinicians.

Heart murmur detection is a more straightforward task than diagnosing a specific disease, which promotes research and development in this field. DL research is based on high-quality data, for which medical professionals and researchers have created rich datasets labeled with murmurs and shared them with the public [8,10,12,119]. Utilizing these datasets and some private datasets, researchers developed several DL models for detecting heart murmurs. PCG signals can be transformed into various types of spectrograms using different transformations and scales, thus converting temporal information into spatial representations. Murmurs display distinct features within these spectrograms, which can be accurately detected. Many models designed for this task primarily use CNN architecture for its effectiveness in processing spatial data representations [32,40,41,44,49,53–55,64,66–68,70–73,75,76,81,82,105–108,111–113]. Other models incorporate more sophisticated NN architectures, such as CNN–RNN hybrids, that leverage both spatial and sequential data processing capabilities [50,69,115]. Other advanced architectures, such as Capsule networks [38], have also been assessed for this task. The most recently developed models also integrate attention mechanisms to enhance feature extraction and improve performance [27,91].

While these models can only identify the presence of heart murmurs and not provide definitive diagnoses, they can play an important role in community screening. This allows people with potential heart problems to be referred to specialists promptly, which is valuable, especially in underprivileged areas with limited medical resources.

#### Valvular heart disease

VHD is a prevalent condition associated with high mortality rates worldwide [120]. Early screening and follow-up are crucial for managing VHD as most patients remain asymptomatic until the advanced stage, resulting in poor prognosis without timely intervention. While echocardiography is the current gold standard for VHD diagnosis [121,122], its cost and requirement for specialized personnel make it impractical for community screening and self-monitoring.

Cardiac auscultation is a simple and cost-effective diagnostic tool for VHD. However, relying solely on auscultation for diagnosis results in low accuracy due to human errors and environmental disturbances [123]. DL techniques have demonstrated superior recognition capabilities compared to humans. Using the Yaseen et al. dataset [11] consisting of 1,000 audio clips of normal heart sounds and 4 VHDs, AS, MS, MR, and MVP, several DL algorithms have been developed to extract heart sound features and train models for VHD diagnosis: First, Yaseen et al. [11] employed MFCCs combined with DWT features as inputs for deep neural network (DNN) classifiers, achieving an accur acy of 92.1%. Second, Ghosh et al. [90] extracted features from the time–frequency matrix of the heart sound recordings and input them into a Deep Layer Kernel Sparse Representation Network classifier, resulting in an overall accuracy of 96.79%. Third, Ding et al. [84] developed a CNN classifier (GoogLeNet) utilizing heart sound time–frequency scalograms based on CWT, achieving an overall accuracy of 97.5%. Fourth, Roy et al. simultaneously constructed a CNN-based inception network, a hybrid CNN–RNN architecture, and an LSTM-based RNN model and compared their performance. The results demonstrated that the CNN-based inception network outperformed the other 2 architectures for heart sound classification [124]. Fifth, Torre-Cruz et al. proposed an approach combining orthogonal nonnegative matrix factorization (ONMF) with CNN architectures in a 3-stage cascade to improve the learning process by identifying optimal ONMF temporal or spectral patterns for accurate VHD detection. This approach increases accuracy by about 45% compared to architectures not using ONMF spectral features [42]. Besides the Yaseen et al. dataset, in the Tromsø study, a population-based prospective study, Waaler et al. collected heart sound recordings and echocardiography results from 2,124 participants. Among them, 408 (19.2%) participants had at least one significant VHD. They developed an LSTM-based RNN model using MFCC-processed heart sound signals as input. This model had excellent discrimination of AS murmurs (area under the curve [AUC] = 0.979), but its performance was mediocre for aortic regurgitation (AUC = 0.634) and MR (AUC = 0.549) [58].

Recently, researchers have worked on constructing automatic “end-to-end” models that do not require signal pre-processing or feature engineering. Also based on the Yaseen et al. dataset [11], Baghel et al. [57] employed a CNN with data augmentation and a Gaussian filter for noise removal, achieving an accuracy of 98.6%. Alkhodari et al. utilized a CNN–RNN model for direct classification using heart sound recordings with one-dimensional wavelet smoothing, resulting in a 99.32% accuracy in a 10-fold cross-validation scheme and 87.31% in external validation based on CinC 2016 dataset [16]. Khan et al. [43] developed a novel Cardi-Net architecture based on a CNN structure to extract discriminative PCG features from the power spectrogram for VHD identification, achieving an accuracy of 98.88%. Jamil and Roy [117] implemented a vision transformer leveraging the self-attention mechanism on PCG signals, further improving the performance in terms of accuracy and computational efficiency. Ma et al. [34] proposed a lightweight CNN architecture, achieving a 92% to 97% reduction in parameters compared to other comparable DL models, with an average accuracy of 98.6% on the Yaseen et al. dataset. In a large-scale study, Chorba et al. [20] trained a CNN model on over 34 h of heart sound recordings from 5,318 patients to detect VHD-related murmurs, yielding a promising performance with a sensitivity of 76.3% and a specificity of 91.4%. Makimoto et al. further improved the interpretability and usability of DL models. They developed a lightweight CNN model to detect severe AS using 1,668 heart sound recordings at 3 auscultation locations from 556 patients. Based on this model, a smartphone application was established, achieving a 95.7% accuracy and 0.93 F1 score. Additionally, they employed Gradient-based Class Activation Maps to identify the specific heart sound features that the DL model focused on when distinguishing the severity of AS [45].

#### Congenital heart disease

CHD is a prevalent cardiovascular disease in children, affecting approximately 0.8% to 1% of the global population [125]. The most common type is left-to-right shunt CHD, including atrial septal defects (ASDs), ventricular septal defects (VSDs), and patent ductus arteriosus (PDA). This condition can cause chronic volume overload, resulting in HF and pulmonary hypertension (PH) [126,127]. Imaging techniques, including echocardiography, magnetic resonance imaging, and computerized tomography, are crucial for CHD evaluation [128]. However, their limited availability and high costs pose challenges, particularly in underdeveloped regions. The delay in diagnosis can lead to irreversible complications and even death [129].

Auscultation plays a vital role in screening and diagnosing CHD as these patients often present with heart murmurs caused by abnormal blood flow through malformed heart structures [130]. However, the accuracy of this method heavily relies on the physicians’ experience, and not all heart murmurs can be accurately identified [131]. To enhance the diagnostic efficiency of heart sound, DL algorithms have been increasingly employed. Wang et al. developed a temporal attentive pooling-convolutional RNN model for VSD detection using heart sound recordings from 51 patients with VSD and 25 healthy individuals, with a sensitivity of 96.0% and a specificity of 96.7% [36]. Huang et al. converted heart sound recordings from 184 participants, including 46 with VSDs, 50 with ASDs, and 88 with a normal heart structure, into bispectrum signals. These signals were then utilized to train an advanced optical coherence tomography network model for heart sound classification. Remarkably, this model outperformed experienced cardiologists in detecting VSD and ASD with an accuracy of 93.4% and 85.3%, respectively [102]. Liu et al. [21] developed a residual convolution RNN model to detect ASD, VSD, PDA, and combined CHD using 884 heart sound recordings from children with left-to-right shunt CHD, with an overall accuracy of 96.79%. Also, for the task of ASD, VSD, and PDA detection, Wang et al. used a fusion of Mel-Frequency Spectral Coefficients and envelope features of heart sounds as input for a locally concatenated fusion approach combined with a CNN based on coordinate attention (LCACNN). This method achieved classification accuracies of 91.78% and 94.79% on the PhysioNet and private databases, respectively [116]. Hassanuzzaman et al. were the first to apply a transformer model to diagnose CHD. They proposed a DL model that classifies raw PCG signals for CHD diagnosis using a one-dimensional CNN combined with an attention transformer. This model, built on raw PCG data from 484 patients, achieved an accuracy of 0.923 and an AUC of 0.964 [23].

In clinical practice, distinguishing between VSD and bicuspid/tricuspid regurgitation through auscultation can be challenging, as both conditions manifest as systolic murmurs in the mitral and tricuspid areas. Gharehbaghi et al. [91] tackled this issue by training a TGNN model to differentiate VSD from valvular regurgitation and healthy subjects using heart sound recordings from 90 individuals, achieving an accuracy of 86.7%. Furthermore, innocent murmurs are present in approximately 50% of children, leading to many unnecessary referrals to pediatric cardiologists [132]. To address the issue, Gharehbaghi et al. [22] developed a TGNN model capable of distinguishing ASD and VSD from valvular regurgitation and innocent murmur using heart sound recordings from 115 children, resulting in an accuracy of 91.6%.

#### Heart failure

HF is a global epidemic with high mortality, affecting over 26 million individuals worldwide, and its prevalence continues to rise due to an aging population [133]. Early detection and timely treatment of HF are crucial for long-term prognosis, as the progression of HF can lead to irreversible myocardial remodeling and functional impairment [134]. Current guidelines outline specific conditions for the diagnosis of HF, including typical symptoms and signs, reduced or preserved LVEF, elevated brain natriuretic peptide levels, and the presence of structural heart disease and diastolic dysfunction [135,136]. However, the symptoms or signs may be nonspecific at the early stages of HF [135,136], and echocardiography and blood biomarker tests are unsuitable for screening purposes.

Heart sounds, as a physiological signal generated by myocardial contraction, can provide direct insights into the mechanical dysfunctions of the heart [137]. However, the heart sounds specific to HF, such as gallop rhythm, usually become apparent at the later stages of HF and require sufficient expertise to identify. With DL algorithms, Gao et al. first proposed an HF screening framework based on a gated recurrent unit (GRU) model, distinguishing between the normal subjects, HF with preserved ejection fraction (HFpEF), and HF with reduced ejection fraction (HFrEF) using heart sounds, with an average accuracy of 98.82% [18]. Wang et al. [17] employed CNN and RNN to build a heart sound diagnostic model that accurately differentiated between normal individuals, HFpEF, and HFrEF, achieving an accuracy of 97.64%. Chen et al. further employed a CNN to classify HF patients’ cardiac function according to the New York Heart Association functional classification. They used the CWT to pre-process heart sound signals into spectra as input for the CNN, achieving an accuracy of 94.34% [77].

In addition to left ventricular systolic dysfunction as mentioned above, diastolic dysfunction is also a common type of HF. Yang et al. developed a CNN model to diagnose left ventricular diastolic dysfunction using heart sounds. They applied data augmentation techniques with deep convolutional generative adversarial networks to enhance the model‘s performance, resulting in an accuracy of 98.7% [37]. Similarly, Zheng et al. proposed a PCG transfer learning-based CatBoost model to detect diastolic dysfunction. They utilized multiple domain-specific deep features extracted from PCG spectrograms using pre-trained CNNs, and fed them into CatBoost for classification and performance comparison, yielding an accuracy of 88.2%. [103]

Once HF is diagnosed, accurately classifying the stages of HF is critical for guiding clinical practice. The American Heart Association (AHA)/ American College of Cardiology (ACC) guidelines define 4 stages of HF (stages A, B, C, and D), ranging from developing HF without symptoms to advanced HF [136]. Zheng et al. utilized heart sound recordings from 275 subjects and employed a deep belief network model that incorporated multi-scale (original signal, sub-sequences, and sub-band signals) and multi-domain (time domain, frequency domain, and nonlinear) features. Their approach achieved 74.3% accuracy in automatically HF staging [19].

#### Coronary artery disease

CAD is a major cause of mortality and morbidity worldwide and substantially burdens the medical system [138]. While coronary angiography is considered the gold standard for CAD diagnosis, its invasive nature and requirement for specialized catheterization laboratories restrict its availability. ECG is another commonly used diagnostic tool, but it has limitations in terms of sensitivity, particularly in stable and asymptomatic patients, and its accuracy highly depends on the expertise of the interpreting physicians [139]. Consequently, there may be a considerable number of undiagnosed CAD cases in underdeveloped regions.

Previous studies have shown that turbulence in stenotic coronary arteries can produce faint high-frequency murmurs [140–142]. However, these faint murmurs are often not discernible during auscultation, and recognizable changes in heart sounds typically occur only after the development of severe structural complications, such as papillary muscle dysfunction, septal perforation, or ventricular dilatation [1]. Given that machine recording can capture faint murmurs, a DL-based diagnostic model shows promise for CAD detection. In 2020, Li et al. developed a CAD detection model using heart sounds. They extracted 110 multi-domain features and MFCCs from the heart sound recordings of 175 subjects. The fusion framework, combining selected multi-domain and DL features, served as input for a CNN classifier, achieving an accuracy of 90.43% [104]. In 2021, Li et al. further improved their approach by developing a multi-input CNN framework that integrated time, frequency, and time–frequency domain deep features from simultaneous ECG and PCG signals of 195 subjects for CAD detection. The model, which combined multi-domain deep features from the 2 modalities, showed high performance in CAD identification, with an accuracy of 96.51% [59]. To address the challenge of limited sample size for training CNN models, Pathak et al. explored transfer learning for CAD detection using heart sounds. They employed a CNN pre-trained on the ImageNet database, consisting of 1 million training images, and transferred its feature representation for CAD detection. Multiple kernel learning was then used to fuse the embeddings of the CNN with handcrafted features, including the heat map of Synchrosqueezing Transform and time-varying Shannon and Renyi Entropy in sub-bands of Synchrosqueezing Transform. Despite having only 40 CAD and 40 normal subjects’ heart sound data, their diagnostic model achieved an accuracy of 89.25% [143]. Ainiwaer et al. recorded heart sounds from 319 patients who underwent coronary angiography, of whom 201 were diagnosed with CAD and 118 were not. They employed state-of-the-art DL architectures (VGG-16, 1D CNN, and ResNet18) to build models for identifying CAD. They found that VGG-16 demonstrated the highest performance, achieving an AUC of 0.834 and outperforming ResNet-18 and CNN-7, which had AUCs of 0.755 and 0.652, respectively [24].

#### Rheumatic heart disease

RHD remains a significant public health issue in developing countries, impacting a minimum of 33 million individuals and contributing to at least 345,000 deaths annually [144]. RHD is caused by an abnormal immune response to beta-hemolytic streptococcal pharyngitis infection and primarily affects the mitral valve. Typically, echocardiography is used to diagnose RHD by evaluating valve morphology and severity of valve dysfunction [145]. However, given the high prevalence of RHD in underdeveloped regions, there is an urgent need to develop a cost-effective screening method for RHD.

The RHD-related damage on the valves disrupts the normal blood flow in the heart chambers and causes murmurs, which presents a possibility of creating a DL-based model for RHD detection using heart sounds. In 2020, Asmare et al. collected 33,453 heart sound clips from 124 RHD patients and 46 healthy individuals. They trained a CNN model using the Mel Spectro-temporal representation of un-segmented PCG, achieving an overall accuracy of 96.1% with 94.0% sensitivity and 98.1% specificity [47].

Compared to intervention after RHD has developed, early detection of subclinical RHD in susceptible populations and providing penicillin for prophylaxis may be a more cost-effective strategy for individuals and healthcare systems [146]. Based on this principle, Ali et al. proposed a study plan to recruit 1,700 children (5 to 15 years) from underprivileged schools in Pakistan and collect clinical data, including heart sound recordings and echocardiograms. They aimed to train a DNN to automatically identify patients with subclinical RHD and definite RHD [25]. This study is currently ongoing, and we look forward to its results.

#### Extracardiac applications

Continuous BP measurements are essential for managing critically ill patients and those undergoing surgery. Invasive intra-arterial cannulation is the gold standard for continuous BP measurement. However, it often causes arterial complications and thrombosis. On the other hand, the cuff BP measurement, the most common noninvasive method, can provide only indirect estimates of systolic and diastolic BP using proprietary formulas, and it does not allow for continuous readings [147]. Heart sounds have shown a close relationship with BP. Previous studies have established a positive correlation between the frequency and amplitude of the second heart sound and BP [148,149]. This relationship can be explained by the mechanical vibrations caused by arterial wall elasticity and blood column inertia [150]. Additionally, the amplitude of the first heart sound has been linked to cardiac contractility [150]. These findings provide a physiological basis for estimating BP using heart sounds with DL techniques. In 2019, Kapur et al. trained an artificial NN model to estimate BP using 737 heart sound recordings from 25 children undergoing continuous BP monitoring via radial artery intra-arterial catheters. The DL model successfully estimated BP, exhibiting a significant correlation with the readings obtained from intra-arterial catheters (*R*^2^ = 0.928 and 0.868 for systolic and diastolic BP, respectively) [151].

PH is a chronic and progressive disease characterized by dyspnea, right HF, and a high mortality risk [152]. The gold standard for diagnosing PH is right heart catheterization, which defines PH as a resting mean PAP of 20 mmHg or higher. However, cardiac catheterization is invasive and thus unsuitable for routine examinations. As an alternative, echocardiography is recommended for estimating PAP, calculated from the maximum peak tricuspid regurgitation velocity using the Bernoulli equation. Nonetheless, echocardiography is operator-dependent and requires optimal acoustic windows and flow tracings to measure PAP accurately, resulting in a delay of up to 2 years between the symptom onset and PH diagnosis [153]. As PAP increases, specific changes occur in heart sounds, including tricuspid regurgitant murmurs, an augmented second heart sound in the pulmonic area, and a third heart sound gallop. Wang et al. utilized the Yaseen et al. dataset [11] and supplemented it with their own heart sound recordings of PH. They translated one-dimensional heart sound signals into 3-dimensional spectrograms using CWT. They employed 10 transfer learning networks to diagnose PH and VHDs, and compared their performance. Their findings revealed that 4 transfer learning networks (ResNet101, DenseNet201, DarkNet19, and GoogleNet) outperformed other models with an accuracy of 98% in detecting PH and 4 VHDs [85]. However, due to the relatively low prevalence of PH, most existing models are trained on small sample sizes, which affects their accuracy. Mi et al. addressed this by inventing an in vitro model of pulmonary circulation, which simulates pulmonary arterial pressure states by adjusting the distal vascular resistance of the pulmonary artery. They collected synchronous PAP data and the vibration sounds of the pulmonary and tricuspid valves. Using a CNN to extract features from the vibration sounds and a Bi-LSTM network to learn time sequence features, they achieved a mean absolute error of 3.403 mmHg in predicting PAP [115].

## Discussion

DL has immense potential in analyzing heart sounds, enabling precise and automated diagnosis of heart conditions. However, this field also presents several challenges and opportunities for further development. The scarcity, quality, and fairness of heart sound data pose significant challenges for DL model training, leading to risks of overfitting and poor generalization. Pre-processing steps, while beneficial for model convergence, may inadvertently filter out valuable information, highlighting the need for further research into their impact on overall performance. Interpretability of DL models remains a concern, as their complexity often makes them difficult to understand and prone to overfitting, which in turn limits their generalization. Future exploration directions include integrating multi-modal data (such as integrating heart sound and ECG data), utilizing wearable devices for continuous monitoring, and enabling convenient real-time diagnostics through smartphone applications.

### Data limitation

#### Data scarcity

The collection and annotation of heart sound data are complex and time-consuming, requiring specialized equipment and trained clinicians. As a result, the quantity of labeled heart sound data is considerably smaller than other medical data types, such as medical images or electronic health records (see Table 2). However, DL models thrive on large-scale datasets to learn and generalize effectively. Insufficient data may result in overfitting, where the model memorizes the available examples instead of learning meaningful features, leading to poor generalization of unseen data. Leveraging transfer learning techniques can be beneficial when faced with limited heart sound data. Pre-training DL models on large-scale datasets from related domains, such as general audio data or medical imaging, can help initialize the model with useful features, enabling it to learn from limited labeled heart sound data more effectively [85,143].

#### Data quality

Another challenge is the quality of heart sound recordings. The acquisition of heart sounds in real-world practice is vulnerable to interference from environmental noise, which may obscure faint murmurs and degrade the quality of recordings [57]. Additionally, the positioning of the stethoscope during data collection can significantly influence the characteristics of recorded heart sounds [1], subsequently affecting the performance of DL models trained on such data. Therefore, it is crucial to investigate and establish standardized and rigorous protocols for heart sound collection to ensure consistent and reliable results.

#### Bias and fairness

Lastly, due to potential demographic biases (such as age, gender, race, etc.) in the training data, DL models may inadvertently learn these biases, resulting in skewed performance across different population groups. For instance, a model trained predominantly on data from a specific demographic group may struggle to generalize to other groups, leading to poorer diagnostic accuracy for those underrepresented in the dataset. To address these challenges, it is essential to ensure that heart sound datasets used for training are representative of the broader population. This involves curating datasets with a balanced distribution of demographic factors to avoid overrepresentation or underrepresentation of any group. Additionally, fairness-aware techniques could be employed during the model development process to detect and mitigate potential biases [154]. Conducting subgroup analyses of model performance across different demographic groups is also essential for identifying and correcting biases, ultimately enhancing the model’s generalizability.

### Pre-processing disadvantage

As previously mentioned, heart sounds are typically pre-processed to extract their frequency information and represent it in a time–frequency format, such as a spectrogram or Mel-spectrogram. This representation is then fed into a DL model, which can identify patterns and features associated with various heart conditions. Although this pre-processing step might make the model converge quicker and incorporate prior knowledge from engineering or human hearing principles, it also risks filtering out valuable information that could benefit the model’s prediction. Numerous studies mentioned above have delved into various signal-processing techniques. However, a comprehensive investigation into the impact of signal processing on the model’s overall performance is still lacking. It is essential to conduct further research and exploration to understand the trade-off between prior knowledge and detailed information in heart sound analysis when employing DL techniques.

### Interpretability shortage

As with the applications of DL in other medical fields, DL in heart sound analysis also faces the challenge of limited interpretability. Since DL models are designed to handle the complexity of large datasets, they often become too complex to fully comprehend or explain. Researchers have explored various methods to interpret DL models used in heart sound analysis. For instance, Cesarelli et al. [155] employ the Gradient-weighted Class Activation Mapping algorithm to highlight the model’s regions of interest in the time–frequency representation image of the PCG transform. Similarly, Wang et al. [156] introduce the SHapley Additivie exPlanations (SHAP) method to interpret the heart sound classification model by evaluating the contribution of each pixel group in the time–frequency representation image. Additionally, Ren et al. [157] examine a heart sound classification network that utilizes attention mechanism and visualizing attention tensors to gain insight into the model’s focus. Despite these advancements, it remains challenging to determine why a model produces a certain result or why it might overlook specific details in the data. Moreover, DL models are susceptible to overfitting, where the model memorizes the specific examples instead of learning meaningful features. As a result, these models are highly specific to a particular dataset and cannot be generalized to other datasets. Therefore, caution is necessary when performing interpretability analysis, as the features identified from an overfitting DL model may not be generalizable.

### Future perspectives

#### Multi-modalities

Integrating different modalities in DL models can uncover hidden patterns and dependencies that might not be apparent when analyzing each modality individually. Shiraga et al. proposed a multi-modal CNN architecture that combines PCG signals and ECG to diagnose severe VHD and ventricular dysfunction. Their study demonstrated that the performance of the multi-modal approach surpassed that of models based solely on PCG or ECG [46]. However, the heart sound and ECG signals used in their study were not collected simultaneously but were recorded separately on the same day. Synchronized heart sound and ECG data can complement the missing information dimensions in a single modality, providing a complete description of the heart’s electro-mechanical activity throughout the cardiac cycle. To our knowledge, only Li et al. have used ML models to process synchronized heart sound and ECG signals [158], and there is currently no DL diagnostic model based on synchronized heart sound and ECG signals. This gap warrants further research and improvement in the future.

Advances in sensor technology and data fusion techniques will be key to realizing the potential of multi-modal diagnostic systems. Future research should focus on developing algorithms that effectively integrate synchronized heart sound and ECG data, as well as other physiological signals like photoplethysmograms and PCGs, to enhance diagnostic accuracy. Exploring the potential of multi-modal data for predictive analytics and personalized medicine will also be a critical area of investigation. Furthermore, ensuring the synchronization of data collection and developing standardized protocols for multi-modal data recording will be essential to facilitate the widespread adoption and clinical validation of these advanced diagnostic models.

#### Wearable devices

Wearable technology is rapidly evolving, offering significant potential for continuous heart sound monitoring and cardiac disease diagnosis. Flexible heart sound sensors, such as those made from fabric materials, provide greater convenience and comfort than traditional rigid sensors, allowing for long-term wear and continuous heart sound recording. These sensors can be integrated into everyday clothing or accessories, enabling unobtrusive monitoring of cardiac health in real time [159].

The continuous data collected by these wearable devices can be processed by DL models to detect anomalies and predict adverse events. For example, patients with HF can benefit from wearable heart sound collection devices that monitor their cardiac function continuously, enabling the early detection of acute exacerbations and timely medical intervention. Despite the potential, there are currently no DL models specifically designed to process long-term heart sound data, highlighting a significant gap in the field. Besides, these models need to be robust and capable of processing noisy and incomplete data, which are common challenges in real-world scenarios. The accumulation of large datasets from wearable devices will be crucial for developing these specialized DL models.

#### Smartphone applications

The enhanced computing power and widespread availability of smartphones make them a powerful platform for deploying DL models for heart sound analysis. Smartphones come equipped with built-in microphones that can capture heart sounds of sufficient quality. In a study conducted by Luo et al. [160] using their smartphone application, more than 80% of users were able to obtain good-quality heart sound recordings, with success rates independent of age, gender, body mass index, and smartphone versions. As mentioned above, Makimoto et al. [45] have developed a smartphone application using a lightweight CNN model to detect severe AS. Smartphone-based diagnostic models offer a convenient way for patients to monitor their health status at home, enabling the early detection of potential diseases and ensuring prompt access to treatment.

In addition, smartphones can be interconnected with wearable health monitoring sensors (e.g., ECGs and photoplethysmographs), which is an ideal platform for the application of multi-modal DL models as mentioned above. In this case, future research should focus on developing sophisticated algorithms capable of analyzing heart sounds alongside other physiological data, creating comprehensive home-based diagnostic tools. Such applications not only democratize healthcare by providing individuals, regardless of their geographic location or socioeconomic status, with the same opportunity to perform disease screenings using their smartphones, but also hold the potential to revolutionize the accessibility and affordability of healthcare.

Moreover, the accuracy of DL models increases with the complexity of their parameter structures, but this also raises computational requirements. Considering the limited computing resources of smartphones, deploying highly accurate and complex models on these devices is challenging. Although cloud computing offers ample resources, it introduces latency due to data transmission. In contrast, edge computing, i.e., using smartphones, can respond to requirements faster than cloud computing, but with limited computing capacities [161]. Therefore, developing a collaborative mechanism between local and cloud computing, allowing deployment systems to balance efficiency and accuracy in data analysis, is a key technical challenge for deploying DL-based diagnostic models on smartphones.

### Conclusion

Cardiac auscultation is a fundamental and essential skill for clinicians, but it requires extensive training and experience to identify and diagnose heart conditions accurately. Nowadays, heart sounds can be easily recorded and analyzed using computers. By combining the traditional signal processing approaches and DL techniques, researchers have made significant progress in detecting a wide range of cardiovascular diseases using heart sounds. While some promising results have been achieved using DL models for diagnosing heart conditions based on PCG data, further research is needed to validate the accuracy and generalizability of these models.
